# A Bumpy Pathway to Stationary-Phase Survival in Bacillus subtilis

**DOI:** 10.1128/mBio.02461-19

**Published:** 2019-10-29

**Authors:** Wayne L. Nicholson

**Affiliations:** a Department of Microbiology and Cell Science, University of Florida, Space Life Sciences Laboratory, Merritt Island, Florida, USA

**Keywords:** *Bacillus subtilis*, mutation, nutrient depletion, stationary phase

## Abstract

Bacillus subtilis cells can mount a number of responses to nutritional deprivation but ultimately either form dormant spores or enter a metabolically quiescent state. In a recent article (mBio 10:e01414-19, https://doi.org/10.1128/mBio.01414-19, 2019), R. Hashuel and S. Ben-Yehuda report on a novel means by which nutrient-starved B. subtilis cells escape from aging (days-old) colonies by accumulating mutations enabling them to continue growth under nutrient-limited conditions. They postulate that such a strategy may be a major factor determining the dynamics of bacterial populations in natural environments.

## Abstract (precis)

Bacillus subtilis cells can mount a number of responses to nutritional deprivation but ultimately either form dormant spores or enter a metabolically quiescent state. In a recent article (mBio 10:e01414-19, https://doi.org/10.1128/mBio.01414-19, 2019), R. Hashuel and S.

## COMMENTARY

The natural environment confronts life with constantly shifting challenges at all scales, from the global down to the microscopic; as a consequence, life has evolved diverse mechanisms for survival when faced with various environmental adversities. The Gram-positive bacterium Bacillus subtilis is arguably one of the best-studied examples of bacterial adaptation to environmental stresses, and workers in the field have a relatively good understanding of the molecular mechanisms that it uses to adapt to physical and chemical stresses, such as high and low temperatures, high osmolarity, anaerobiosis, oxidative agents, toxic metals, etc. (reviewed in reference 1). A hallmark of B. subtilis adaptability, and a subject that has been of particular interest to researchers since the beginnings of microbiology, is the organism’s response and adaptation to nutritional stress. One of the earliest observations in microbiology by Cohn and Koch in the 19th century, working independently on B. subtilis and Bacillus anthracis, respectively, was that these rod-shaped bacteria formed dormant, optically refractile, and highly resistant spores in nutrient-depleted cultures and that such spores could germinate and resume growth when supplied with fresh nutrients (2). Since these early observations, B. subtilis has become one of the premier model systems for studying both the sporulation process and other systems activated by the transition from exponential growth to the stationary phase in response to nutrient deprivation (1). A large number of adaptations to nutrient starvation, in addition to the initiation of sporulation, have been uncovered and studied intensively in B. subtilis; examples are the synthesis and excretion of polymer-degrading enzymes, antibiotics, and secondary metabolites, motility and chemotaxis, genetic competence, and biofilm formation (1, 3, 4). However, upon prolonged starvation, B. subtilis cells were understood either to differentiate into dormant spores or to enter a metabolically reduced state, the stationary phase.

In a recent article, Hashuel and Ben-Yehuda (5) describe and characterize a novel and seemingly counterintuitive response of B. subtilis cells to nutrient restriction. As they observed colonies aging over a number of days, they noted the appearance of smaller microcolonies distributed over the surface of each primary colony. Individual strains isolated from these microcolonies displayed altered morphologies and were dubbed “morphomutants.” Whole-genome sequencing of several of these morphomutants revealed mutations in genes associated with sporulation initiation, transition state functions, or basic cellular processes (transcription, translation, replication, or metabolism). They postulate that mutations in these genes allowed the morphomutant strains to escape quiescence and to continue growth, presumably by feeding off alternative nutrient sources or the nutrients released by the lysis of their neighbors. Thus, mutations allowing continued growth in the stationary phase may be seen as an alternative pathway to surviving nutrient restriction. Viewed as such, B. subtilis morphomutants are reminiscent of the growth advantage in stationary phase (GASP) phenotype of Escherichia coli, which has also been associated with mutations in metabolic genes or the transition state sigma factor *rpoS* (6). The authors point out that, although morphomutants can immediately escape a nutrient-restricted environment via continued growth, the fact that they have lost from their repertoire the ability to sporulate or otherwise enter metabolic quiescence seems to bode poorly for their long-term survival in nature.

**FIG 1:**
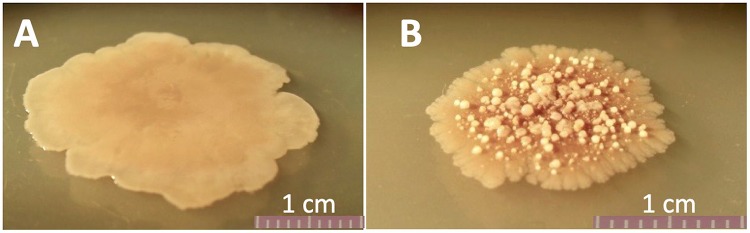
Five-day old colonies of wild-type ancestral strain WN628 (A) and evolved “bumpy” mutant strain WN657 (B).

Upon viewing the colony images, I was immediately reminded of a long-term evolution experiment that my graduate student Heather Maughan had conducted nearly 20 years ago (7). In that experiment she noted that within only a few hundred generations of evolution, B. subtilis cells underwent an increase in their spontaneous mutation rate, and variants with altered colony morphologies began to appear in the evolving populations. I was struck by the similarity in appearance of one of Heather’s colony variants, which we called “bumpy” (Fig. 1), to that of Hashuel and Ben-Yehuda’s aging colonies with their emerging morphomutants. This happened years before the advent of next-generation genome sequencing; we simply made note of these bumpy mutants and stored them away in the freezer, where they remain uncharacterized to this day. It is gratifying to see that a similar phenomenon has been observed by Hashuel and Ben-Yehuda, and I salute the thorough and elegant experiments by which they have elucidated the mechanism resulting in morphomutants.
